# Defining Surrogate Endpoints for Clinical Trials in Severe Falciparum Malaria

**DOI:** 10.1371/journal.pone.0169307

**Published:** 2017-01-04

**Authors:** Atthanee Jeeyapant, Hugh W. Kingston, Katherine Plewes, Richard J. Maude, Josh Hanson, M. Trent Herdman, Stije J. Leopold, Thatsanun Ngernseng, Prakaykaew Charunwatthana, Nguyen Hoan Phu, Aniruddha Ghose, M. Mahtab Uddin Hasan, Caterina I. Fanello, Md Abul Faiz, Tran Tinh Hien, Nicholas P. J. Day, Nicholas J. White, Arjen M. Dondorp

**Affiliations:** 1 Mahidol-Oxford Tropical Medicine Research Unit, Faculty of Tropical Medicine, Mahidol University, Bangkok, Thailand; 2 Charles Darwin University, Darwin, Northern Territory, Australia; 3 Centre for Tropical Medicine and Global Health, Nuffield Department of Clinical Medicine, University of Oxford, Oxford, United Kingdom; 4 Global Health Division, Menzies School of Health Research, Darwin, Australia; 5 University College, Oxford, United Kingdom; 6 Department of Clinical Tropical Medicine, Faculty of Tropical Medicine, Mahidol Unversity,Bangkok,Thailand; 7 Oxford University Clinical Research Unit. Hospital for Tropical Diseases, Ho Chi Minh City, Vietnam; 8 Department of Medicine, Chittagong Medical College Hospital, Chittagong, Bangladesh; 9 Kinshasa School of Public Health, University of Kinshasa, Kinshasa, Democratic Republic of the Congo; 10 Malaria Research Group, Dev Care Foundation, Dhaka, Bangladesh; Centro de Pesquisas Rene Rachou, BRAZIL

## Abstract

**Background:**

Clinical trials in severe falciparum malaria require a large sample size to detect clinically meaningful differences in mortality. This means few interventions can be evaluated at any time. Using a validated surrogate endpoint for mortality would provide a useful alternative allowing a smaller sample size. Here we evaluate changes in coma score and plasma lactate as surrogate endpoints for mortality in severe falciparum malaria.

**Methods:**

Three datasets of clinical studies in severe malaria were re-evaluated: studies from Chittagong, Bangladesh (adults), the African ‘AQUAMAT’ trial comparing artesunate and quinine (children), and the Vietnamese ‘AQ’ study (adults) comparing artemether with quinine. The absolute change, relative change, slope of the normalization over time, and time to normalization were derived from sequential measurements of plasma lactate and coma score, and validated for their use as surrogate endpoint, including the proportion of treatment effect on mortality explained (PTE) by these surrogate measures.

**Results:**

Improvements in lactate concentration or coma scores over the first 24 hours of admission, were strongly prognostic for survival in all datasets. In hyperlactataemic patients in the AQ study (n = 173), lower mortality with artemether compared to quinine closely correlated with faster reduction in plasma lactate concentration, with a high PTE of the relative change in plasma lactate at 8 and 12 hours of 0.81 and 0.75, respectively. In paediatric patients enrolled in the ‘AQUAMAT’ study with cerebral malaria (n = 785), mortality was lower with artesunate compared to quinine, but this was not associated with faster coma recovery.

**Conclusions:**

The relative changes in plasma lactate concentration assessed at 8 or 12 hours after admission are valid surrogate endpoints for severe malaria studies on antimalarial drugs or adjuvant treatments aiming at improving the microcirculation. Measures of coma recovery are not valid surrogate endpoints for mortality.

## Introduction

Falciparum malaria remains an important cause of mortality, with an estimated global death toll of 438 000 (range 236 000–635 000) in 2015 [1]. Case fatality in severe falciparum malaria remains high, despite optimal antimalarial treatment with parenteral artesunate. Overall case fatality in African children has been reported as 4.2%, but is highly dependent on the case-definition of severe malaria and the extent of organ failure [2]. In children with cerebral malaria without multi-organ involvement the mortality is 6%; this increases to 43% when patients also have renal dysfunction and metabolic acidosis [3, 4]. A large range of adjuvant therapies have been studied in clinical trials, but none has proven to be beneficial [5–7]. However, most of these trials have been underpowered. This means that some therapies might have been unjustly rejected as having no beneficial effect. Sufficiently large trials in severe malaria with a mortality endpoint, however, may not be feasible or affordable. For example, in order to show a 10% relative change in mortality with a new therapy in pediatric severe malaria, starting from an (artesunate) treated mortality of 9% [8], 30,000 patients need to be randomized. For a larger difference of 30%, which poses very high expectations on a new intervention, still around 3,000 patients would be needed. With the current global reduction in malaria transmission, this would imply that only few interventions and adjunctive therapies can be studied satisfactorily at any moment in time. This problem is not unique to the study of severe malaria. For instance, in trials on bacterial sepsis, several authors have argued for more frequent use of surrogate endpoints and organ specific outcome measures [9].

Severe malaria is a multi-organ disease. In both adult and pediatric patients coma depth and severity of metabolic acidosis have strong prognostic significance [10]. Indices of improvement in these parameters could be potential surrogate endpoints for mortality when evaluating novel therapies. Indeed, clinical studies in African children with severe malaria show that plasma lactate concentrations reduce more slowly in patients with a fatal outcome from the disease [11]. However, it has not been explored how different measures of improvement in metabolic acidosis or coma are related to mortality and how much of a treatment effect on mortality can be explained by these parameters. The validation of biomarkers as surrogate endpoints is challenging. It is not sufficient that a biomarker correlates with the true endpoint to make it an appropriate surrogate endpoint. According to Prentice, there is the requirement for the surrogate variable to capture any relationship between the treatment and the true endpoint [12]. In order to validate fully a surrogate, meta-analysis of multiple trials of drugs in the same class are required measuring the surrogate and the clinical benefit endpoint [13]. Since this level of validation is prohibitively restrictive, a hierarchy of endpoints with different levels of evidence supporting them has been proposed [13]. In the current study we evaluated which dynamic variables have the strongest predictive value for mortality, using three independent large datasets from studies in severe falciparum malaria [8, 14–21]. In addition, we assessed the proportion of the effect on mortality explained by the different surrogate endpoints.

## Materials and Methods

### Patients and data

The datasets from studies organized by our research groups used for the analysis were: 1. Serial Glasgow Coma Scale (GCS) and Blantyre Coma Score (BCS) data assessed 6 hourly from the AQUAMAT (ISRCTN50258054) randomized controlled trial comparing parenteral artesunate with quinine treatment in African children with severe malaria [8]. 2. Serial lactate data (assessed at 4, 8, 12 and 24 hours after enrolment) from Vietnamese adult patients with severe malaria in the AQ study comparing artemether with quinine treatment. [21]; and 3. Serial GCS (assessed 6 hourly) and lactate (assessed 6 hourly) data from adult patients with severe malaria enrolled in studies at Chittagong Medical College Hospital (CMCH), Chittagong, Bangladesh between 2005 and 2012. The Bangladeshi patients were enrolled in observational and treatment studies including studies on levamisole, timing of enteral feeding, N-acetylcysteine and paracetamol [15, 18, 20, 22]. These randomized clinical trials were monitored internally as well as by an outside monitor. Data quality of the variables included in the current analyses were guaranteed through data validation checks. Details on the study population and methods have been published previously. A published adjusted analysis for the AQ study has shown a mortally benefit in favor of artemether over quinine [21]. In line with current guidelines, hyperlactatemia was defined as a plasma lactate concentration above 4 mmol/L [5]. Coma depth was assessed as GCS or BCS in preverbal children, with coma defined as a GCS<11 or BCS <3 [5].

### Surrogate endpoints assessed

We explored a range of dynamic measures, denoting the change over time in coma score or plasma lactate concentration, for their association with mortality or antimalarial treatment arm. The four measures assessed were: change from admission at a specified time, expressed as either the absolute (1) or relative change (2), the slope of the log-linear change of the variables over time (3), and time to normalization till a normal coma score or a plasma lactate ≤2 mmol/L (4).

### Analysis of variable dynamics summarized as absolute change, relative change and slope of the log-time curve

To calculate the absolute change in plasma lactate or coma score, the lactate concentration or coma score at the time of follow-up was subtracted from the lactate concentration or coma score measured at enrolment. To calculate the relative change, the absolute change at the follow-up timepoints of interest were divided by the measurement at enrolment. The specified timepoints used to evaluate coma depth were 6, 12, 18 and 24 hours after enrollment. Plasma lactate levels were assessed at 4, 8, 12, and 24 hours in the AQ study, or at 6, 12, 18, and 24 hours from enrolment in the Chittagong dataset. In case of an early fatal outcome of the disease, the worst value of the variable observed in the dataset was imputed as the follow-up values. Imputed data were not used in models predicting mortality or for calculation of the treatment effect explained.

The majority of patients showed a log-linear relationship between coma score or plasma lactate concentration versus time since start of treatment. Regression using individual patient data was used to calculate the slope of the log lactate or log coma score vs time for each patient. The slope over different timespans was assessed. The timespan used were up to 6, 12, 18 and 24 hours for coma score. For plasma lactate, the timespans used were up to 4, 8, 12, and 24 hours after enrolment in the AQ study, or up to 6, 12, 18, and 24 hours in the Chittagong dataset. A generalized estimating equation (GEE) model was used to estimate the effect of mortality or treatment group on lactate clearance.

For each endpoint three logistic regression models were constructed using: (a) the baseline value of the parameter assessed; (b) the dynamic measure of the parameter; and (c) a combination of the baseline value of the parameter and the dynamic measure of interest. To explore whether a surrogate endpoint measure was an independent predictor for mortality, a likelihood ratio test was used to compare the model (a) only using the baseline value with (c) the model including both baseline and surrogate endpoint. The area under receiver operation characteristic curve (AUROC) for the surrogate endpoints regarding mortality prediction and the optimum cut off point were calculated from model (b). The adjusted mean values of these endpoints were also compared by mortality outcome and by treatment allocation using analysis of covariance (ANCOVA), adjusting for baseline lactate or coma score. The proportion of treatment effect explained (PTE) by the surrogate measure under consideration was calculated as: PTE = (α_1_ - β_1_)/ β_1_, with α_1_ and β_1_ defined by the logistic regression models: Logit (mortality) = α_0_ + α_1_(treatment group) and Logit (mortality) = β_0_ + β_1_(treatment group) + β_2_(surrogate measure). PTE values range from 0 to 1, with 1 indicating a perfect surrogate and 0 indicating lack of any surrogacy. A bootstrap method with 1,000 replicated samples was used for constructing the PTE 95% confidence interval (CI). The calculated confidence interval can be expected to be wide because of residual variability [23].

### Analysis of parameter dynamics summarized as time to event

For assessment of the time to normalization, a normal lactate concentration was defined as ≤2 mmol/l and coma recovery as improvement to a GCS of 15 or a BCS of 5. Data were assessed for both prediction of disease outcome and surrogate endpoint by treatment allocation. Mann Whitney U tests were used to compare median clearance or recovery times. The Cox proportional hazards model was used to calculate the hazard ratio (HR) for recovery or clearance time. Competing risks regression was used to calculate the sub-distribution hazard ratio (SDHR) for recovery or clearance time. The comparison between groups was expressed as the cumulative proportion of patients recovered to a normal concentration of plasma lactate (≤2 mmol/l) or maximum coma score, per person hour Death and loss to follow-up were considered as competing events for lactate clearance or coma recovery. HR and SDHR were calculated both unadjusted, and adjusted for baseline lactate or coma score.

In all analyses, a p-value of <0.05 was considered to be statistically significant. Statistical analyses were performed using STATA version 13.0 (StataCorp LP, Texas, USA).

## Results

### Baseline characteristics

In the AQUAMAT study, a total of 1,940 out of 5,425 enrolled children presented with coma (GCS<11 or BSC<3), of whom 785 had 6 hourly coma scores available for evaluation. In the Chittagong studies 137/318 patients presented with coma. Hyperlactataemia (>4 mmol/L) on enrollment was observed in 173/560 patients in the AQ study and in 124/318 patients in Chittagong studies. The baseline characteristics and survival outcomes of patients represented in the different datasets are shown in S1 Table. Plasma lactate and coma score on admission were strong predictors for mortality. In the AQ study, the odds ratio for mortality was 1.3 (95%CI 1.1 to 1.4, p<0.001) per unit (mmol/L) increase in plasma lactate concentration. In the AQUAMAT study the odds ratio for mortality was 0.66 (95%CI 0.57 to 0.77, p<0.001) per unit increase in GCS and 0.67 (95%CI 0.44 to 1.03, p = 0.07) per unit increase in BCS.

### Change in plasma lactate concentration as surrogate endpoint for mortality

In the AQ study, mortality in patients presenting with hyperlactataemia was 38/173 (22%). In this group, mortality was lower in patients treated with artemether (14/81, 17%) compared to quinine (24/92, 26%; p = 0.16). A log-linear slope could be fitted to the plasma lactate longitudinal clearance data in 103/173 (60%) of hyperlactataemic patients.

Table 1 compares the different dynamic measures for plasma lactate regarding their performance as surrogate endpoints in the AQ dataset. The AUROC for mortality prediction of the different measures (absolute change, relative change or slope of the log-linear time curve), ranged from 0.66 to 0.74, with overlapping confidence intervals for the different measures. In all cases, the surrogate endpoint predicted mortality independent of the baseline lactate as indicated by a significant likelihood ratio test (p<0.05) comparing the model with baseline only vs the model with baseline and the surrogate measure.

**Table 1:** AUROC, cut-points, summary statistic and Proportion Treatment Effect Explained (PTE) for measures of change in plasma lactate assessed at various times after start of treatment in the AQ study. AM, Artemether; QN, Quinine; Se, Sensitivity; Sp, Specificity.

Surrogate measurement	Hour 4	Hour 8	Hour 12	Hour 24
**AUROC, (95% CI)**				
N	172	158	159	152
Absolute change	0.67 (0.56,0.77)	0.66 (0.51–0.80)	0.71 (0.58–0.85)	0.67 (0.52–0.82)
Relative Change	0.68 (0.58, 0.78)	0.66 (0.52–0.80)	0.72 (0.60–0.84)	0.68 (0.52–0.83)
Log slope	0.68 (0.58–0.78)	0.71 (0.60–0.82)	0.74 (0.63–0.84)	0.74 (0.63–0.85)
**Cut-point (Se,Sp)**				
Absolute change	-0.5 (0.61, 0.65)	-1 (0.65, 0.64)	-1.1 (0.69, 0.70)	-2.5 (0.72, 0.54)
Relative Change	-9 (0.66, 0.65)	-23 (0.69, 0.59)	-27 (0.69, 0.67)	-45 (0.67, 0.60)
Log slope	-.02 (0.66, 0.65)	-0.02 (0.71, 0.66)	-.02 (0.71,0.71)	-.02 (0.68, 0.67)
**Mean value of surrogate, adjusted for baseline**				
**Absolute change (Mean, 95% CI)**				
***Mortality***				
Alive	-1.2* (-1.6,- 0.8)	-2.2*(-2.6, -1.8)	-2.9* (-3.3, -2.4)	-3.6* (-4, -3.3)
Death	0.8 (0.1, 1.5)	0.4 (-0.5, 1.3)	1 (-.04, 2)	-1 (-1.9,.-01)
***Treatment***				
AM	-1.3* (-1.8, -0.88)	-1.7* (-2.86, -0.54)	-2.08* (-3.35, -0.80)	-1.92* (-3.47, -0.38)
QN	-0.22 (-0.69, 0.24)	0.67 (-0.41, 1.76)	0.38 (-0.82, 1.57)	0.50 (-1, 2)
**Relative Change (Mean, 95% CI)**				
***Mortality***				
Alive	-16* (-21, -10)	-27* (-33, -21)	-36* (-42, -29)	-46* (-52, -40)
Death	8 (-2, 18)	6 (-7, 20)	12 (-2, 27)	-6 (-22, 10)
***Treatment***				
AM	-18* (-25, -11)	-23* (-36, -10)	-28* (-43, -14)	-30* (-48, -12)
QN	-3 (-10, 3)	5 (-8, 17)	-1 (-14, 13)	1 (-16, 18)
**Log slope (Mean, 95% CI)**				
***Mortality***				
Alive	-0.06* (-0.07, -0.04)	-0.05* (-0.06, -0.04)	-0.05* (-0.06, -0.04)	-0.04* (-0.05, -0.03)
Death	0.01 (-0.02, 0.03)	0.01 (-0.01, 0.03)	0.01 (-0.01, 0.03)	0 (-0.01, 0.02)
***Treatment***				
AM	-0.06* (-0.08, -0.04)	-0.05* (-0.06, -0.04)	-0.04 (-0.05, -0.03)	-0.03 (-0.04, -0.02)
QN	-0.02 (-0.04, -0.01)	-0.03 (-0.04, -0.01)	-0.03 (-0.04, -0.02)	-0.03 (-0.04, -0.02)
**PTE (95% CI)**				
Absolute change	0.56 (-2.59, 4.47)	0.73 (-6.07, 6.73)	0.77 (-7.92, 7.19)	-0.22 (-0.54, 2.42)
Relative Change	0.52 (-2.59, 4.64)	0.81 (-6.85, 7.46)	0.75 (-9.39, 6.70)	-0.33 (-0.67, 2.09)
Log slope	0.53 (-2.42, 4.19)	0.59 (-3.11, 4.96)	0.47 (-2.76, 4.59)	0.26 (-1.90, 2.83)

* P-value < 0.05.

**Fig 1:**
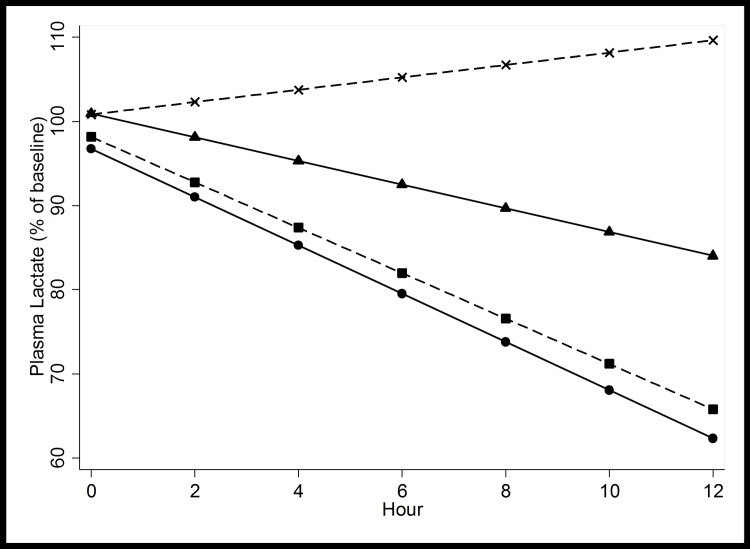
Generalized estimating equations model (GEE) for the change in plasma lactate according to antimalarial treatment (artemether designated with a circle or quinine designated with a triangle) and patient outcome (survivors designated with a square or fatal cases designated with a X). GEE choses the optimal values to construct the best fitted linear slope. This includes the baseline plasma lactate value, which can thus deviate slightly from 100%.

Table 1 shows that absolute and relative change in plasma lactate concentration were significantly different between treatment groups at all timepoints assessed, with larger decreases observed with artemether. The absolute reduction in lactate was significantly greater in the artemether treated patients at 4, 8 and 12 hours but not at 24 hours. Fig 1 illustrates the change in lactate concentration over time between antimalarial treatment and patient outcome as fitted by GEE.

The proportion of the treatment effect explained (PTE) was calculated for all method-timepoint combinations (Table 1). The maximum PTE values were observed at plasma lactate concentrations assessed at 8 and 12 hours: at 8 hours the PTE value of the absolute or relative change in plasma lactate was 0.73 and 0.81 respectively, at 12 hours these values were 0.77 and 0.75.

### Time to normalization of lactate

In hyperlactatemic patients (plasma lactate >4 mmol/L) enrolled in the AQ study, plasma lactate returned to normal (≤2 mmol/L) in 115/173 (66%) patients, with a median (IQR) time to lactate clearance of 48 (24 to 72) hours. Of the remaining 34%, normalization did not occur for 33/173 (19%) due to death of the patient and for 25/173 (14%) because of loss of follow-up. After normalization of plasma lactate, 5/115 (4%) died during the subsequent hospitalization period, whereas the remainder recovered. The majority of patients with a fatal course (33/38, 87%) never achieved normalization of plasma lactate concentration.

The median time to lactate clearance was not different between survivors and fatal cases. However, using Cox proportional hazards model, or using competing risks regression, both the hazard ratio and sub-distribution hazard ratio were significantly lower than 1, indicating that time to lactate clearance was prognostic for outcome. Median lactate clearance times were not different between treatment arms) and the hazard ratio or the sub-distribution hazard ratio for lactate clearance was not significantly different. After adjusting for the plasma lactate concentration at enrolment, the sub-distribution hazard ratio for treatment with artemether on lactate clearance was significantly greater than 1, indicating a greater cumulative incidence of lactate clearance in the artemether arm as compared to the quinine arm (Table 2).

**Table 2:** Cumulative proportion recovered to normal plasma lactate concentration per person hour in patients randomized to treatment with either artemether (AM) or quinine (QN) in the AQ study. HR, Hazard Ratio; SDHR, Sub-Distribution Hazard Ratio.

	HR	Baseline adj.HR	SDHR	Baseline adj. SDHR
Outcome				
Alive (n = 135)				
Death (n = 38)	0.41* (0.17–1.0)		0.11* (0.04–0.26)	
Treatment				
AM (n = 81)	1.21 (.84–1.74)	1.34 (0.91–1.96)	1.33 (0.96–1.84)	1.63* (1.17–2.29)
QN (n = 92)				

* P-value < 0.05.

### Validation in the chittagong dataset

The prognostic ability of the different dynamic measures for plasma lactate over time was validated using the Chittagong studies as an independent dataset. In the 124 Bangladeshi patients with hyperlactatemia, median (IQR) enrollment plasma lactate was 6.5 mmol/L (5.0 to 9.2 mmol/L) compared to 6.0 mmol/L (4.8 to 8.3 mmol/L) in the AQ study. Overall mortality was 46/124 (37%), of which 18/46 (39%) occurred within 12 hours and 31/46 (67%) within 24 hours. In the Chittagong dataset, the median (IQR) time to lactate clearance was 36 (24 to 48) hours in the 70 patients clearing plasma lactate to ≤2 mmol/L. Of these latter patients 8/70 (11%) died after normalization of plasma lactate. As in the AQ dataset, the majority of in-hospital deaths (38/46 or 83%) occurred before normalization of plasma lactate.

At all of the timepoints assessed, the relative change in lactate was an independent predictor of mortality at all timepoints (6, 12, 18, and 24 hours), as assessed by comparison of the logistic regression models containing baseline lactate alone or baseline lactate and the surrogate endpoint (S2 Table). The absolute and relative change in plasma lactate concentration were only a significant independent predictor for survival at timepoints 12, 18 and 24 hours. These timepoints were also significant when assessing the slope of the log-linear change in lactate. Since all Bangladeshi patients were treated with parenteral artesunate, no comparison between different antimalarial treatments could be made.

### Change in coma score as surrogate endpoint for mortality

Of the 785 patients with cerebral malaria that were analyzed from the AQUAMAT study, 229 pre-verbal children were assessed by BCS and the remainder 556 patients by GCS. Overall mortality was 115/785 (15%). In 52/115 (45%) patients, death occurred within the first 12 hours and in 73/115 (64%) within 24 hours after admission.

Tables 3 and 4 compare the different dynamic measures for coma depth as surrogate endpoints in the AQUAMAT dataset. A log-linear slope could be fitted to the data in 359/829 (43%) of comatose patients. The AUROC denoting the prognostic value for patient survival ranged from 0.75 to 0.87 for the different measures: absolute change, relative change or slope of the coma scores over time. All measures predicted mortality independent of baseline lactate (p<0.05).

**Table 3:** AUROC, cut-points, summary statistic and Proportion Treatment Effect Explained (PTE) for measures of change in Blantyre Coma Score assessed at various times after start of treatment in the AQUAMAT study. AS, Artesunate; QN, Quinine; Se, Sensitivity; Sp, Specificity.

Surrogate measurement	Hour 6	Hour 12	Hour 18	Hour 24
**AUROC (95% CI)**				
N	220	208	194	174
Absolute change	0.78 (0.73–0.84)	0.78 (0.71–0.85)	0.83 (0.76–0.90)	0.82 (0.73–0.91)
Relative Change	0.77 (0.72–0.83)	0.76 (0.69–0.84)	0.82 (0.75–0.89)	0.80 (0.69–0.91)
Log slope	0.77 (0.72–0.83)	0.79 (0.73–0.85)	0.84 (0.79–0.90)	0.87 (0.82–0.92)
**Cut-point (Se, Sp)**				
Absolute change	1 (0.64, 0.88)	2 (0.62,0.87)	2 (0.72, 0.83)	2 (0.77, 0.79)
Relative Change	8 (0.64, 0.88)	8 (0.72, 0.64)	16 (0.72, 0.83)	17 (0.77, 0.79)
Log slope	.01 (0.64,0.89)	.01 (0.73, 0.73)	.01 (0.75, 0.78)	0 (0.8, 0.8)
**Mean value of surrogate, adjusted for baseline**				
**Absolute change (Mean, 95% CI)**				
***Mortality***				
Alive	1.4* (1.2, 1.6)	2* (1.8, 2.2)	2.3* (2.1, 2.5)	2.5* (1.3,2.7)
Death	0 (-0.3–0.4)	0.3 (-0.2,1)	0.4 (-0.1, 0.8)	0.4 (-0.1, 0.9)
***Treatment***				
AS	1.1 (0.8, 1.3)	1.6 (1.3, 1.9)	1.7 (1.4, 2)	1.7 (1.3, 2)
QN	1.1 (0.9, 1.4)	1.4 (1.1, 1.7)	1.6 (1.2, 1.9)	1.7 (1.3, 2)
**Relative Change (Mean, 95% CI)**				
***Mortality***				
Alive	13* (11,14)	18* (16, 20)	20* (19, 22)	22* (20, 24)
Death	0 (-3, 3)	3 (-1, 6)	3 (-1, 7)	4 (-1, 8)
***Treatment***				
AS	9 (7,11)	14 (11, 17)	15 (12, 18)	14 (11, 18)
QN	10 (8,12)	12 (9, 15)	14 (11, 17)	15 (12, 18)
**Log slope (Mean, 95% CI)**				
***Mortality***				
Alive	0.02* (0.02, 0.02)	0.02* (0.02, 0.02)	0.02* (0.02,0.02)	0.02* (0.02, 0.02)
Death	0 (0,0)	0 (0, 0.01)	0 (0,0)	0 (0,0)
***Treatment***				
AS	0.01 (0.01, 0.02)	0.01 (0.01, 0.02)	0.01 (0.01,0.02)	0.01 (001, 0.02)
QN	0.01 (0.01,0.02)	0.01 (0.01, 0.02)	0.01 (0.01, 0.02)	0.01 (0.01,0.02)
**PTE (95% CI)**				
Absolute change	-0.07 (-2.80, 2.58)	0.39 (-4.18, 5.75)	-0.59 (-1.76, 1.59)	-0.9 (-6.39, 5.31)
Relative Change	-0.08 (-2.87, 2.58)	0.36 (-4.07, 5.29)	-0.59 (-1.68, 1.47)	-0.86 (-6.20, 4.98)
Log slope	-0.14 (-4.08, 3.53)	0.26 (-2.92, 3.70)	0.10 (-3.18, 2.90)	0.11 (-3.23, 3.26)

* P-value < 0.05.

**Table 4:** AUROC, cut-points, summary statistic and Proportion Treatment Effect Explained (PTE) for measures of change in Glasgow Coma Scale assessed at various times after start of treatment in the AQUAMAT study. AS, Artesunate; QN, Quinine; Se, Sensitivity; Sp, Specificity.

Surrogate measurement	Hour 6	Hour 12	Hour 18	Hour 24
**AUROC (95% CI)**				
N	545	524	508	487
Absolute change	0.76 (0.70–0.83)	0.82 (0.74–0.89)	0.82 (0.76–0.88)	0.87 (0.80–0.93)
Relative Change	0.75 (0.69–0.82)	0.80 (0.71–0.88)	0.78 (0.69–0.87)	0.84 (0.76–0.93)
Log slope	0.75 (0.69–0.82)	0.83 (0.76–0.89)	0.85 (0.78–0.91)	0.87 (0.82–0.93)
**Cut-point (Se,Sp)**				
Absolute change	1 (0.60, 0.81)	1 (0.80, 0.73)	2 (0.78, 0.77)	2 (0.84, 0.81)
Relative Change	10 (0.60, 0.81)	10 (0.80, 0.73)	25 (0.77, 0.71)	38 (0.79, 0.81)
Log slope	.02 (0.60, 0.81)	.01 (0.79, 0.78)	.01 (0.79, 0.80)	.01 (0.87, 0.78)
**Mean value of surrogate, adjusted for baseline**				
**Absolute change (Mean, 95% CI)**				
***Mortality***				
Alive	2* (2,2)	4* (3, 4)	5* (4, 5)	5* (5, 6)
Death	-1 (-1, 0)	0 (-1, 1)	0 (-1, 1)	0 (-1, 1)
***Treatment***				
AS	2 (1,2)	3 (2, 3)	4 (3, 4)	4 (4, 5)
QN	2 (2,2)	3 (3, 4)	4 (4, 5)	5 (4, 5)
**Relative Change (Mean, 95% CI)**				
***Mortality***				
Alive	32* (28, 35)	54* (49, 59)	70* (65, 75)	80* (75, 85)
Death	-9 (-20, 2)	-11 (-27, 5)	-6 (-25, 14)	-15 (-36, 5)
***Treatment***				
AS	25 (20, 30)	41 (34, 48)	55 (48, 63)	63 (55, 71)
QN	30 (24, 35)	49 (43, 56)	62 (55, 69)	70 (62, 77)
**Log slope (Mean, 95% CI)**				
***Mortality***				
Alive	0.04* (0.03, 0.04)	0.04* (0.03, 0.04)	0.03* (0.03, 0.04)	0.03* (0.03, 0.04)
Death	-0.02 (-0.03, -0.01)	-0.02 (-0.03, -0.02)	-0.02 (-0.03, -0.01)	-0.02 (-0.03, -0.01)
***Treatment***				
AS	0.03 (0.02, 0.04)	0.03 (0.02,0.03)	0.03 (0.02, 0.03)	0.03 (0.02, 0.03)
QN	0.04 (0.03, 0.04)	0.03 (0.03, 0.04)	0.03 (0.03, 0.03)	0.03 (0.02, 0.03)
**PTE (95% CI)**				
Absolute change	-1.22 (-5.58, 9.34)	-3.53 (-7.52, 8.04)	-2.72 (-7.71, 6.90)	-3.65 (-11.52, 11.76)
Relative Change	-0.93 (-7.41, 8.08)	-2.93 (-5.43, 5.89)	-2.14 (-4.95, 4.40)	-3.20 (-9.67, 9.02)
Log slope	-1.24 (-6.89, 8.81)	-1.67 (-10.74, 9.42)	-1.54 (-16.64, 10.21)	-1.83 (-15.13, 14.56)

* P-value < 0.05.

In this subset of patients from the AQUAMAT study, mortality with artesunate was 13%, compared to 16% with quinine treatment (p = 0.24). None of the dynamic measures for change in coma depth over time showed a difference between antimalarial treatment arm. The proportion of the treatment effect explained (PTE) by the dynamic coma measure was <0 for most measures explored, implying that these dynamic measures for coma depth do not explain differences in survival between antimalarial treatments (Tables 3 and 4). Only the absolute or relative change in BCS at 12 hours, or the slope of the BCS score over time assessed up to 12 hours provided positive PTE values of 0.39, 0.36 and 0.26, respectively (Table 3).

### Time to normalization of coma score

The median (IQR) time to normalization of the coma score in patients from the AQUAMAT study was 24 (12 to 48) hours. A total of 648/785 (83%) patients recovered from coma, after which another 4/648 (1%) of the children died. For comparison, in the Chittagong dataset, the median (IQR) time to coma clearance was 42 (24–48) hours. In the latter adult patients, a total of 73/137 (53%) recovered and after recovery, another 4/73 (6%) of the patients died.

When using the Cox proportional hazards model, or using competing risks regression, both the adjusted and unadjusted hazard ratio and sub-distribution hazard ratio for coma recovery were significantly different from 1 indicating these measures are prognostic for survival. However, analysis by treatment group showed that the median coma recovery times were not different and the unadjusted and adjusted sub-distribution hazard ratios were not significantly different from 1 (Table 5).

**Table 5:** Cumulative proportion recovered to maximal coma score per person hour in patients randomised to treatment with either artesunate (AS) or quinine (QN) in AQUAMAT. HR, Hazard Ratio; SDHR, Sub-Distribution Hazard Ratio.

		HR	Baseline adj. HR	SDHR	Baseline adj. SDHR		HR	Baseline adj. HR	SDHR	Baseline adj. SDHR
Outcome										
Alive (n = 670)										
Death (n = 115)	GCS	0.06*(0.02–0.18)		0.02* (0.005–0.05)		BCS	.06*(.02-.20)		0.02* (0.005–0.06)	
Treatment										
AS (n = 388)		0.90 (0.72–1.03)	0.89 (0.75–1.06)	0.91 (0.78–1.08)	0.90 (0.76–1.06)		1.03 (0.76–1.39)	1.03 (0.76–1.39)	1.14 (0.87–1.51)	1.15 (0.87–1.51)
QN (n = 397)										

* P-value < 0.05.

### Validation in the chittagong dataset

The prognostic ability of the different endpoints describing change in coma score was validated in the independent dataset from Chittagong, Bangladesh. In the adult patients with cerebral malaria mortality was 45/137 (33%), of whom 17/45 (38%) died within 12 hours and 27/45 (60%) within 24 hours. As in the AQUAMAT dataset, the absolute change, relative change and slope of the GCS over time were significantly different between survivors and fatal cases, with an AUROC for the relative change in GCS at 12 hours of 0.78 (95%CI 0.64 to 0.92) in the Bangladeshi patients compared to 0.80 (95%CI 0.71 to 0.88) in children with cerebral malaria enrolled in AQUAMAT. At all timepoints assessed (6, 12, 18, and 24 hours), the absolute and relative change in time and the slope in GCS over time were all independent prognosticators for mortality, as assessed by comparison of the logistic regression models containing baseline lactate alone or baseline lactate and the surrogate endpoint as independent variables (S3 Table).

## Discussion

In this study potential surrogate endpoints for intervention studies in severe falciparum malaria were evaluated. Improvement in plasma lactate concentrations or coma score, assessed at a range of timepoints within the first 24 hours of admission, were strongly prognostic for survival, independent of the baseline value. However, whereas faster clearance of plasma lactate was predictive of the treatment effect on mortality of artemether compared to quinine, faster coma recovery was not. Changes in plasma lactate assessed at any timepoint up to 24 hours after admission performed similarly in terms of prognostic significance for mortality. The relative change in plasma lactate at 8 or 12 hours after admission showed the highest value for the treatment effect explained (PTE), suggesting adequate performance as a surrogate endpoint. Compared to the absolute change in plasma lactate concentration, the relative change was less dependent on the baseline value. The slope of the log-lactate time plot has the advantage that more measure points are included in the assessment. However, the decline in plasma lactate was not log linear over time in all cases, reducing its utility. Time to plasma lactate clearance was only prognostic for survival when the competing risk of death prior to lactate clearance was taken into consideration. Since severe malaria has a high mortality, censoring this most severely ill group of patients who died prior to lactate clearance will bias the results [24]. A difference in the cumulative incidence of lactate clearance between treatment groups was observed when competing risks methodology was used and the subdistribution hazard ratio was calculated. Thus, provided the right methodology is applied, time to lactate clearance is a suitable alternative surrogate endpoint. Measures of change in lactate concentration have the disadvantage that only around 50% of the adult or pediatric patients with severe malaria will present with metabolic acidosis; other patients can have a poor prognosis related to coma or acute kidney injury [3, 4]. In addition, some therapies may aim to improve survival through a mechanism not expected to affect lactate clearance. Examples include interventions aiming to reduce aspiration pneumonia in comatose patients, or restricted fluid management aiming to prevent pulmonary oedema, where other endpoints are warranted.

Despite coma recovery time having been used as an endpoint in multiple studies in severe malaria [18, 25, 26], our analysis showed that improvement in coma score in patients presenting with cerebral malaria was not correlated with improved survival in the AQUAMAT dataset comparing artesunate versus quinine treatment. The reduction in morality in favour of artesunate treatment would not have been detected if coma clearance were used as the surrogate endpoint. This observation was also made in the large treatment study, the AQ study, comparing parenteral artemether with quinine in adult patients with severe malaria, showing better survival with artemether in the adjusted analysis, despite the coma recovery time being prolonged [21]. The survival benefit of parenteral artemisinins over quinine in cerebral malaria is therefore clearly not mediated by a faster resolution of coma. This finding does not imply that coma is not associated with mortality in patients with cerebral malaria; deep coma is associated with death due respiratory arrest [27, 28] and death due to secondary consequences of coma such as aspiration pneumonia or airway obstruction, a particular risk in resource limited settings [18]. Rather, the survival benefit of artesunate over quinine in patients with cerebral malaria may be through improving mortality related to other factors than coma itself. As proposed previously, the longer coma recovery time with the more effective antimalarial drug could result from higher survival of patients that would otherwise have died [29, 30]. Although the rate of coma recovery is not a suitable surrogate endpoint measure for studies on antimalarials in severe malaria, it could still be an effective outcome measure for interventions targeting factors associated with coma more directly.

An implicit prerequisite for a mortality surrogate endpoint is an essential role of the outcome variable in the causal chain of events leading to a fatal outcome, or a directly related measure of the variable (such as lactate for metabolic acidosis). Because of this causal relationship, an improvement in mortality is directly related to an improvement in the surrogate maker. However, the relative contributions of different disease mechanisms to death from severe malaria are still debated [31, 32]. An important underlying pathophysiological mechanism involved in acidosis and coma, and to a lesser extent in renal failure, is microvascular sequestration of parasitized red blood cells causing a compromised microcirculation falciparum malaria [14, 31, 33]. If microcirculatory obstruction is one of the pivotal mechanisms, improvement of plasma lactate concentrations through improvement of the microcirculation will be associated with an improvement in mortality. Interventions aiming at improving microcirculatory flow then reduce simultaneously plasma lactate and mortality. Following artesunate treatment the rapid killing of ring stage parasites prevents their further maturation and sequestration in the microcirculation and this is thought to be a main contributor to the improvement in case fatality [8]. It should be noted that lactate in itself is not toxic and is for instance used in fluid therapy as Ringers’ lactate. In a similar fashion, dichloracetate may be effective at reducing plasma lactate levels [34], however, it seems unlikely to be an effective adjuvant reducing the underlying causes of tissue hypoxia and mortality. Several of the current experimental adjuvant or supporting treatments are aiming at an improvement of the microcirculation in severe malaria, including nitric oxide donors [35, 36], and compounds affecting cytoadhesion and sequestration [15, 37]. Our results suggest that improvement in blood or plasma lactate concentrations over time is an adequate endpoint for trials evaluating these interventions. An alternative potential dynamic marker is Angiopoietin-2 (Ang-2), which was not evaluated in the current study. This marker was recently found to be associated with endothelial dysfunction and temporal changes in Ang-2 have shown to be strongly predictive for fatal disease[38]. Change in Ang-2 concentrations over time has been used as a surrogate endpoint in intervention trials with inhaled nitric oxide aimed at improving the microcirculation, and faster normalization of Ang-2 was associated with improved outcome [39]. This endothelial biomarker thus deserves additional evaluation. Outcome measures such as coma recovery time or lactate clearance time have been previously analyzed using methods not considering the impact of the competing risk of death in the analysis. Censoring patients who die before coma recovery or lactate clearance may introduce bias. Use of competing risks methodology would avoid this Failure to consider this may falsely result in the rejection or acceptation of new therapies. Good performance of a surrogate endpoint also does not invalidate the importance of other parallel or related processes causing death, which could also be targeted for intervention. For instance, children with cerebral malaria might suffer a respiratory arrest and die because of cerebral herniation, seizures, brainstem dysfunction due to microvascular obstruction, sedative medication or airways obstruction. Clinical intervention studies should attempt to establish the cause of death in cerebral malaria, and ideally categorize these as cerebral function-related causes versus secondary complications of coma. The latter, as with many of the complications of severe malaria, should be addressed by interventions improving supportive care for severe malaria, rather than targeting malaria specific mechanisms of disease. Lastly, in most circumstances, use of surrogate endpoints to provide evidence in phase 2 clinical studies, does not abolish the need for phase 3 studies with death or disability free survival as primary endpoint. Previous studies in cardiovascular medicine have shown that overreliance on surrogate endpoints can ignore unrecognized side effects of a medication that increase mortality[40]. However in the case of addressing syndromes that are severe but relatively rare, such as pulmonary oedema in adults with severe malaria, a mortality endpoint study might not be feasible, and a different outcome measure may have to be accepted.

In conclusion, the relative change in plasma lactate concentration assessed at 8 or 12 hours after start of therapy is an appropriate endpoint for intervention trials aiming to improve mortality in severe falciparum malaria, if improvement of tissue perfusion is considered in the causal chain of events. The relative change in coma score is strongly associated with survival, but is a poor surrogate endpoint for studies on antimalarials. Alternative surrogate endpoints may be appropriate for interventions targeting more specific organs or aspects of the disease.

## Supporting Information

S1 Table Baseline patient characteristic of the three datasets. (DOCX)

S2 Table AUROC, cut-points, and summary statistic for measures of change in plasma lactate assessed at various times after start of treatment in the Chittagong dataset. AS, Artesunate; QN, Quinine; Se, Sensitivity; Sp, Specificity; * P-value < 0.05.
(DOCX)

S3 Table AUROC, cut-points and summary statistic for measures of change in Glasgow Coma Scale at various times after start of treatment in the Chittagong dataset. AS, Artesunate; QN, Quinine; Se, Sensitivity; Sp, Specificity; * P-value < 0.05.
(DOCX)
