# Supplementary material for: Defining Surrogate Endpoints for Clinical Trials in Severe Falciparum Malaria
Source: PLoS One. 2017 Jan 4;12(1):e0169307. doi: 10.1371/journal.pone.0169307 (PMC5215574; doi:10.1371/journal.pone.0169307)
Supplement: S1 Table — (DOCX) [file pone.0169307.s001.docx]

**S1 Table. Baseline patient characteristic of the three datasets**

|  | **Artemether** | **Quinine** |
| --- | --- | --- |
| **AQ study** |  |  |
| N (%) | 81 (47%) | 92 (53%) |
| Male(%) | 58 (45%) | 70 (55%) |
| Median age (IQR) | 27 (21, 40) | 31 (24, 43) |
| Median baseline lactate | 6.8 (5.3, 9.7) | 5.4 (4.5, 7.1) |
|  |  |  |
|  | **Artesunate** | **Quinine** |
| **AQUAMAT study** |  |  |
| N (%) | 388 (49%) | 397 (51%) |
| Male(%) | 193 (48%) | 216 (54%) |
| Median age in month (IQR) | 37 (24, 58) | 38 (25, 60) |
| Median baseline BCS | 2 (1, 2) | 2 (1, 2) |
| Median baseline GCS | 7 (6, 9) | 7 (6, 8) |
|  |  |  |
|  | **Artesunate** | **Quinine** |
| **Chittagong study** |  |  |
| **Lactate** |  |  |
| N (%) | 96 (77%) | 28 (23%) |
| Male(%) | 76 (79%) | 19 (68%) |
| Median age (IQR) | 35 (24, 45) | 36 (21, 45) |
| Median baseline lactate | 6.2 (4.9, 9.4) | 6.9 (5.3, 8.9) |
|  |  |  |
| GCS | **Artesunate** | **Quinine** |
| N (%) | 83 (61%) | 54 (31%) |
| Male(%) | 63 (63%) | 37 (37%) |
| Median age (IQR) | 32 (23, 50) | 29 (22, 40) |
| Median baseline GCS | 8 (6, 10) | 7 (4, 9) |
