# Supplementary material for: Defining Surrogate Endpoints for Clinical Trials in Severe Falciparum Malaria
Source: PLoS One. 2017 Jan 4;12(1):e0169307. doi: 10.1371/journal.pone.0169307 (PMC5215574; doi:10.1371/journal.pone.0169307)
Supplement: S2 Table — AS, Artesunate; QN, Quinine; Se, Sensitivity; Sp, Specificity; * P-value < 0.05. (DOCX) [file pone.0169307.s002.docx]

**S2 Table. AUROC, cut-points, and summary statistic for measures of change in plasma lactate assessed at various times after start of treatment in the Chittagong dataset. AS, Artesunate; QN, Quinine; Se, Sensitivity; Sp, Specificity; * P-value < 0.05**

| Surrogate measurement | Hour 6 | Hour 12 | Hour 18 | Hour 24 |
| --- | --- | --- | --- | --- |
| **AUROC (95% CI)** |  |  |  |  |
| N | 100 | 69 | 63 | 94 |
| Absolute change | 0.53 (0.41, 0.65) | 0.48 (0.33, 0.64) | 0.56 (0.38, 0.74) | 0.63 (0.48, 0.78) |
| Relative Change | 0.60 (0.48, 0.71) | 0.56 (0.41, 0.72) | 0.68 (0.53, 0.84) | 0.70 (0.56, 0.84) |
| Log slope | 0.60 (0.48, 0.72) | 0.63 (0.51, 0.75) | 0.67 (0.56, 0.78) | 0.73 (0.63, 0.84) |
| **Cut-point (Se,Sp)** |  |  |  |  |
| Absolute change | -1.59 (0.51, 0.56) | -3.35 (0.510, 0.42) | -2.62 (0.53, 0.61) | -2.97 (0.58, 0.59) |
| Relative Change | -4 (0.65, 0.56) | -42 (0.58, 0.65) | -49 (0.68, 0.61) | -48 (0.67, 0.74) |
| Log slope | -0.07 (0.61, 0.67) | -0.05 (0.61, 0.68) | -0.03 (0.61, 0.71) | -0.03 (0.70, 0.70) |
| **Absolute change (Mean, 95% CI)** | |  |  |  |
| ***Mortality*** |  |  |  |  |
| Alive | -2.77 (-3.42, -2.12) | -3.57* (-4.48, -2.67) | -4.50* (-5.45, -3.55) | -4.93* (-5.42, -4.43) |
| Death | -1.92 (-2.68, -1.16) | -1.48 (-2.74, -0.22) | -1.18 (-2.60, 0.24) | -2.70 (-3.52, -1.87) |
| ***Treatment*** |  |  |  |  |
| AS | -2.53 (-3.06, -2.0) | -1.07 (-2.65, 0.51) | -0.27 (-2.12, 1.58) | -0.87 (-2.4, 0.65) |
| QN | -1.77 (-2.98, -0.57) | 2.90 (-1.48, 7.27) | 4.11 (-0.52, 8.76) | -0.79 (-3.84, 2.27) |
| **Relative Change (Mean, 95% CI)** | |  |  |  |
| ***Mortality*** |  |  |  |  |
| Alive | -36 (-44, -28) | -42* (-53, -32) | -54* (-66, -442) | -61* (-68, -55) |
| Death | -23 (-33, -14) | -16 (-31, -1) | -14 (-32, 5) | -29 (-40, -18) |
| ***Treatment*** |  |  |  |  |
| AS | -32 (-39, -26) | -6 (-29, 18) | 1 (-26, 28) | -12 (-34, 9) |
| QN | -21 (-36, -6) | 42 (-23, 108) | 52 (-17, 119) | -5 (-48, 38) |
| **Log slope (Mean, 95% CI)** |  |  |  |  |
| ***Mortality*** |  |  |  |  |
| Alive | -0.09 (-0.10, -0.07) | -0.07* (-0.08, -0.05) | -0.06* (-0.08, -0.05) | -0.06* (-0.06, -0.04) |
| Death | -0.06 (-0.08, -0.04) | -0.03 (-0.05, -0.02) | -0.03 (-0.04, -0.01) | -0.01 (-0.03, 0) |
| ***Treatment*** |  |  |  |  |
| AS | -0.08 (-0.09, -0.06) | -0.05 (-0.06, -0.04) | -0.04 (-0.06, -0.03) | -0.04 (-0.05, -0.03) |
| QN | -0.06 (-0.09, -0.03) | -0.07 (-0.01, -0.04) | -0.07 (-0.09, -0.04) | -0.04 (-0.06, -0.02) |
|  |  |  |  |  |
