# Supplementary material for: Defining Surrogate Endpoints for Clinical Trials in Severe Falciparum Malaria
Source: PLoS One. 2017 Jan 4;12(1):e0169307. doi: 10.1371/journal.pone.0169307 (PMC5215574; doi:10.1371/journal.pone.0169307)
Supplement: S3 Table — AS, Artesunate; QN, Quinine; Se, Sensitivity; Sp, Specificity; * P-value < 0.05. (DOCX) [file pone.0169307.s003.docx]

**S3 Table. AUROC, cut-points and summary statistic for measures of change in Glasgow Coma Scale at various times after start of treatment in the Chittagong dataset. AS, Artesunate; QN, Quinine; Se, Sensitivity; Sp, Specificity; * P-value < 0.05**

| Surrogate measurement | Hour 6 | Hour 12 | Hour 18 | Hour 24 |
| --- | --- | --- | --- | --- |
| **AUROC (95% CI)** |  |  |  |  |
| N | 89 | 57 | 63 | 79 |
| Absolute change | 0.66 (0.55, 0.77) | 0.79 (0.66, 0.92) | 0.77 (0.63, 0.90) | 0.79 (0.65, 0.94) |
| Relative Change | 0.65 (0.54, 0.77) | 0.78 (0.64, 0.92) | 0.77 (0.63, 0.91) | 0.79 (0.64, 0.94) |
| Log slope | 0.66 (0.54, 0.77) | 0.72 (0.61, 0.83) | 0.73 (0.64, 0.84) | 0.76 (0.67, 0.85) |
| **Cut-point (Se,Sp)** |  |  |  |  |
| Absolute change | 0 (0.76, 0.32) | 0 (0.87, 0.50) | 1 (0.76, 0.67) | 1 (0.73, 0.77) |
| Relative Change | 0 (0.77, 0.32) | 0 (0.87, 0.50) | 10 (0.76, 0.67) | 10 (0.73, 0.77) |
| Log slope | 0 (0.76, 0.32) | 0.01 (0.64, 0.79) | 0.01 (0.72, 0.73) | 0 (0.74, 0.73) |
| **Absolute change (Mean, 95% CI)** | |  |  |  |
| ***Mortality*** |  |  |  |  |
| Alive | 1.5* (0.7, 2.2) | 2.4* (1.4, 3.4) | 3* (1.7, 4.0) | 3.8* (2.9, 4.7) |
| Death | -1 (-1.8, 0.2) | -1.1 (-2.6, 0.4) | -0.5 (-2.1, 1.0) | -0.7 (-2.5, 1.0) |
| ***Treatment*** |  |  |  |  |
| AS | 0.6 (-0.3, 1.4) | 0 (-1.2, 1.2) | 0.5 (-0.7, 1.7) | 0.7 (-0.4, 1.9) |
| QN | 0.9 (-0.2, 1.9) | 1.1 (-0.3, 2.4) | 1 (-0.5, 2.4) | 2.4 (1, 3.8) |
| **Relative Change (Mean, 95% CI)** | |  |  |  |
| ***Mortality*** |  |  |  |  |
| Alive | 25* (13,46) | 42* (24, 61) | 52* (30, 75) | 65* (50, 87) |
| Death | -8 (-31, 15) | -7 (-34, 20) | 4 (-28, 36) | 5 (-30, 40) |
| ***Treatment*** |  |  |  |  |
| AS | 15 (-3, 33) | 10 (-10, 30) | 18 (-5, 40) | 22 (1, 43) |
| QN | 19 (-3, 41) | 18 (-5, 42) | 20 (-8, 47) | 42 (16, 68) |
| **Log slope (Mean, 95% CI)** |  |  |  |  |
| ***Mortality*** |  |  |  |  |
| Alive | 0.03* (0.01, 0.04) | 0.04* (0.02, 0.05) | 0.03* (0.02, 0.05) | 0.03* (0.02, 0.04) |
| Death | -0.02 (-0.04, 0) | -0.01 (-0.03, 0.01) | -0.01 (-0.02, 0.01) | -0.01 (-0.02, 0.01) |
| ***Treatment*** |  |  |  |  |
| AS | 0.01 (-0.01, 0.03) | 0.02 (0, 0.03) | 0.02 (0.01, 0.03) | 0.01 (0, 0.02) |
| QN | 0.01 (-0.01, 0.04) | 0.02 *0.01, 0.04) | 0.02 (0.01, 0.03) | 0.02 (0.01, 0.03) |
|  |  |  |  |  |
